# Spinal Cord Stimulation for Spinal Cord Injury-Related Pain: A Pilot Study

**DOI:** 10.3390/brainsci14121173

**Published:** 2024-11-22

**Authors:** Alexander Alamri, Meredith MacDonald, Alaa Al-Mohammad, Lucia Ricciardi, Michael G. Hart, Erlick A. Pereira

**Affiliations:** 1 Institute of Neurosciences and Cell Biology, City St. George’s, University of London, London WC1E 7HU, UK; aalamri@sgul.ac.uk; 2 Department of Neurosurgery, St. George’s University Hospital, London SW17 0QT, UK; meredith.macdonald@stgeorges.nhs.uk (M.M.); michael.hart8@nhs.net (M.G.H.); epereira@sgul.ac.uk (E.A.P.)

**Keywords:** spinal cord stimulation, chronic pain, spinal cord injury, high-frequency stimulation, neuropathic pain, pain management, quality of life, neurostimulation therapy, traumatic SCI, non-traumatic SCI

## Abstract

Background: Spinal cord stimulation (SCS) has emerged as an effective treatment for managing chronic pain that is unresponsive to traditional therapies. While SCS is well documented for conditions like failed back surgery syndrome (FBSS) and complex regional pain syndrome (CRPS), its effectiveness in managing pain related to spinal cord injuries (SCI) is less studied. This study aims to assess the efficacy of SCS in alleviating SCI-related pain and improving patients’ quality of life, filling a gap in the existing literature. Methods: This cohort study included 15 adult patients with traumatic and non-traumatic SCIs, treated between 2016 and 2022. Patients received SCS implants after either a trial or direct implantation. Pain levels were assessed using visual analog scale (VAS) scores, while quality of life was evaluated using the EuroQol five-dimensional (EQ-5D) scale. The SCS devices were implanted at different spinal levels, with various stimulation protocols applied, including high-frequency stimulation (10 kHz). Results: In patients with traumatic SCI, the mean VAS score decreased from 8.6 to 4.5, with 71% reporting more than 50% pain relief. Non-traumatic SCI patients experienced a reduction from 8.5 to 2.5, with all showing more than 50% pain relief. EQ-5D scores improved in both groups. A 49% reduction in pain medication usage was also observed, though one patient required revision surgery due to an adverse event. Conclusions: SCS significantly reduces pain and improves quality of life for SCI patients, particularly with high-frequency protocols. While promising, further research is needed to optimize patient selection and stimulation parameters for better long-term outcomes.

## 1. Introduction

Chronic pain is defined as persistent pain that lasts for more than three to six months, extending beyond the expected period of healing [1]. Unlike acute pain, which is a temporary response to injury or illness, chronic pain can persist long after the initial cause has been treated or resolved. It affects an individual’s physical, emotional, and social well-being, leading to a significant decrease in quality of life [2]. Chronic pain may arise from various conditions such as nerve damage, arthritis, or injury, and is notoriously difficult to manage effectively over time.

Conventional therapies for chronic pain often follow a multimodal approach. These treatments include medications such as over-the-counter analgesics or prescription drugs like opioids and neuropathic agents (e.g., gabapentin or pregabalin). While medications can provide temporary relief, their long-term use—especially with opioids—carries risks of side effects, dependency, and tolerance [3,4,5]. Other standard treatments include physical therapy to improve mobility and strength, nerve block injections to disrupt pain signals, and psychological therapies like cognitive-behavioral therapy to address the mental health impacts of chronic pain. Despite this broad range of options, many patients continue to experience debilitating pain that is resistant to conventional therapies, especially if they suffer from more complex chronic pain conditions.

A particularly complex form of chronic pain is neuropathic spinal cord injury (SCI)-related pain, which is common among individuals who have sustained an injury to the spinal cord. SCI pain can affect between 40 and 53% of patients and is often debilitating [6,7]. SCI pain is typically classified into two main types: nociceptive pain (caused by damage to tissues, muscles, or bones) and neuropathic pain (caused by damage to the spinal cord itself or nerves). In SCI, neuropathic pain is classified based on its relation to the neurological level of injury (NLI). “At-level” neuropathic pain arises from damage to the spinal cord or nerve roots and is perceived at the NLI or within three dermatomes below it. This pain is characterised by sensory deficits, allodynia (pain from non-painful stimuli), hyperalgesia (increased sensitivity to painful stimuli), and descriptions such as burning, tingling, pins and needles, sharp, shooting, squeezing, painful cold, or electric shock-like sensations [8]. Conditions like syringomyelia, which can cause such pain, are also included in this category. “Below-level” neuropathic pain occurs more than three dermatomes below the NLI due to spinal cord damage and shares similar characteristics; it can affect individuals with both complete and incomplete injuries. If pain extends from below-level into the at-level region without distinct separation, it is considered below-level pain. Neuropathic pain not caused by the SCI but by other conditions—such as diabetic neuropathy, postherpetic neuralgia, or carpal tunnel syndrome—is classified as “other” neuropathic pain.

This type of pain is particularly difficult to treat with conventional therapies and significantly impacts the patient’s quality of life, adding emotional and social stress to their physical limitations [9]. Neurosurgical ablative procedures such as dorsal horn and dorsal root entry zone ablation are invasive and irreversible, but they can provide pain relief for certain SCI patients. Neurorestorative treatments are increasingly being explored as crucial interventions for managing pain and enhancing function in patients with traumatic or non-traumatic SCI [10]. These therapies aim to repair or support damaged neural structures, promoting recovery and alleviating chronic pain. Cell-based approaches, such as mesenchymal precursor cell infusions, have shown potential for improving outcomes in spinal cord degeneration and related pain conditions. Additionally, neuromodulation techniques, including spinal cord stimulation (SCS) and selective dorsal rhizotomy, provide alternative pain management solutions, with varying levels of supporting evidence ranging from observational studies to randomised trials. However, the efficacy of these treatments, particularly in combination with conventional methods, requires validation through more comprehensive, high-level evidence to ensure their safety and effectiveness [10].

SCS offers a promising alternative for patients whose pain does not respond to traditional treatments. SCS is a neuromodulation therapy that involves implanting a device near the spinal cord to deliver mild electrical pulses. These pulses interfere with the transmission of pain signals to the brain, effectively reducing the sensation of pain. The SCS system consists of electrodes implanted near the spinal cord and a small pulse generator placed under the skin, usually in the lower back or abdomen. The electrical pulses can be adjusted by the patient using an external remote control, allowing for tailored pain management.

SCS is a well-established and significant treatment option for managing refractory chronic pain that is unresponsive to conservative therapies, medications, and other interventions [11]. Efficacy and safety are critical factors in evaluating treatments for chronic pain. A comprehensive review by Cameron investigated the use of SCS for managing chronic pain in the trunk and limbs [12]. The author assessed 68 studies that met specific efficacy criteria, involving a total of 3679 patients grouped by pain indication. Additionally, 51 studies satisfied all safety inclusion and exclusion criteria. The findings demonstrated that SCS provides positive, long-term symptomatic relief for conditions such as refractory angina pain, severe ischemic limb pain due to peripheral vascular disease, peripheral neuropathic pain, and chronic low-back pain. This suggests that SCS is generally a safe and effective treatment for various chronic neuropathic conditions.

For patients, SCS works somewhat like a “mask” for pain signals—similar to how white noise can drown out background sounds. The electrical stimulation modifies how the brain perceives pain by altering the pain signals as they travel through the spinal cord [13]. Unlike many drug therapies, SCS is minimally invasive, adjustable, and reversible, making it an attractive option for patients with chronic, refractory pain. Commonly used for conditions such as failed back surgery syndrome, complex regional pain syndrome, and neuropathic pain, SCS provides an effective, long-term solution for many who have exhausted conventional treatment options [14]. Over time, the technology behind neuromodulation devices has improved considerably, making them increasingly sophisticated and reliable [15]. SCS has been particularly effective in treating mixed neuropathic pain, complex regional pain syndrome (CRPS), failed back surgery syndrome (FBSS), and peripheral neuropathy. In addition to these indications, SCS has also been trialled for use in pain management after spinal cord injuries [16]. Despite the promising potential of SCS in the management of SCI-related pain, there remains a limited amount of literature on its efficacy in this specific context, and the literature provides mixed results [17]. A major review of 27 studies involving SCS in SCI patients [18] found limited success, with most reporting low success rates of 30–40%. The majority of these studies focused on chronic pain, not specifically SCI-related pain, and were retrospective with inconsistent patient data. However, the largest study, which included 101 SCI patients, reported a 34% success rate over two years. While some studies showed modest success, particularly for incomplete SCI patients with steady pain, the overall efficacy of SCS in SCI patients remains unproven.

The aim of this study is to retrospectively evaluate the outcomes of SCS as a therapeutic intervention for managing central neuropathic pain associated with SCI. Chronic pain following SCI can significantly impact quality of life, and conventional treatments often provide limited relief. By analysing patient outcomes following SCS implantation, we seek to assess its efficacy in reducing pain, improving functional capacity, and enhancing overall patient satisfaction. This retrospective review will also identify factors that influence success rates, including injury characteristics, patient demographics, and the duration of SCS therapy, to better inform clinical decisions and optimise future treatment strategies for SCI-related chronic pain.

## 2. Methods

### 2.1. Study Design

This was a retrospective study designed to evaluate the efficacy of SCS in the management of chronic neuropathic pain among patients with traumatic and non-traumatic SCI. The chart review process encompassed a time frame from March 2023 to July 2023.

### 2.2. Study Population

All patients operated at St George’s Hospital, London, between February 2016 and July 2023 were included. The chart review process involved identifying eligible patients based on predefined inclusion and exclusion criteria that were those routinely applied in clinical practice for SCS patient selection, including a diagnosis of traumatic and non-traumatic SCI, presence of chronic pain, and the refractory nature of the pain to other medical and interventional treatments.

The appropriateness of SCS for this cohort of patients was assessed using European Guidelines [19]. For chronic low-back and/or leg pain, the appropriateness for SCS increases in patients with a history of previous spinal surgery, particularly when the pain is predominantly in the leg or has a significant neuropathic component. Additional factors that support the use of SCS include a partial or temporary response to prior treatments such as nerve root blocks, transcutaneous electrical nerve stimulation (TENS), radiofrequency ablation, or neuropathic medications. In cases where there are anatomical abnormalities, such as scar tissue or iatrogenic nerve lesions, the appropriateness for SCS is also heightened. For patients with symptoms mimicking CRPS, the appropriateness for SCS is higher when the pain is primarily neuropathic or ischemic, often due to vasomotor disturbances, and when the pain has a limited spread. Positive responses to prior interventions, such as nerve blocks or neuropathic medications, further support the use of SCS in these patients.

### 2.3. Inclusion Criteria

Patients were included if they were aged 18 or older, had a confirmed diagnosis of either traumatic or non-traumatic spinal cord injury (SCI) with chronic neuropathic pain persisting for more than 6 months, and had shown inadequate response to standard pain management treatments. All patients had an established ASIA score to document the level of spinal cord injury and were psychologically screened to ensure suitability for SCS. Additionally, patients were medically cleared for SCS implantation [19].

### 2.4. Exclusion Criteria

Exclusion criteria included active systemic or local infections, anatomical contraindications preventing safe lead placement, untreated coagulopathies, and primary nociceptive pain without a neuropathic component. Patients with significant untreated psychiatric disorders, pregnancy, device incompatibility (e.g., pacemakers), or those unable to provide informed consent were also excluded.

### 2.5. Classification of SCI

To classify the severity of SCI among patients in this study, the American Spinal Injury Association (ASIA) Impairment Scale (AIS) was employed. The ASIA scale is a standardised tool widely used in clinical practice to assess both motor and sensory function, enabling a comprehensive evaluation of the neurological impact of SCI. The scale evaluates motor strength in key muscle groups and sensation across 28 dermatomes on each side of the body, testing both light touch and pinprick sensation. Each dermatome and muscle group is scored to reflect the degree of preserved function below the level of injury.

This classification method is integral in guiding clinical decision making and predicting rehabilitation outcomes. For the purposes of this study, the ASIA scale was used to classify patients with SCI, enabling a structured approach to evaluating the appropriateness of SCS as a treatment option based on their neurological status. The detailed classifications are presented in Table 1.

All patients provided informed consent prior to the SCS procedures. All patients underwent an SCS implantation following a methodology described elsewhere [20]. All patients underwent magnetic resonance imaging (MRI) and/or computed tomography (CT) scans to assess the anatomical characteristics of their spinal cord injury and the condition of surrounding tissues in order to plan the lead placement during the SCS procedure.

### 2.6. Data Collection

Data collected included demographic information, clinical history, procedural details, and post-procedural outcomes. These data were retrieved from the hospital electronic records; all data were de-identified and recorded in a standardized format for subsequent statistical analysis.

### 2.7. Clinical Assessment and Outcome Measures

Outcome measures were primarily focused on pain relief, changes in quality of life, and any adverse events, with follow-up data extending up to January 2024.

Pain: The pain ratings were measured using a visual analog scale (VAS), which are widely recognised for their ability to gauge pain intensity [21]. The VAS scale ranges from 0 = “No Pain” to 10 = “Worst Possible Pain”.

Pain medication use (including the use of opioids, gabapentinoids, and other analgesics) and their dosage were collected.

Quality of life: the Euroqol five-dimensional (EQ-5D) instrument was employed to measure health-related quality of life, evaluating five dimensions, including mobility, self-care, usual activities, pain/discomfort, and anxiety/depression [22].

Adverse events: any adverse effects, including infections, lead migrations, or discomfort at the implantation site, were recorded. Adjustments to stimulation parameters were made based on patient feedback and clinical assessment to ensure optimal coverage of pain regions. Any complications or need for revision surgery were recorded.

All measurements were collected at baseline, 3 months, and 12 months post-SCS implantation, allowing for sufficient rehabilitation time and stabilisation of the intervention outcomes.

### 2.8. Statistical Analysis

Descriptive statistics were used to summarise the demographic and clinical data of the study population. Continuous variables, such as age, were presented as means, standard deviations (SD), and ranges, while categorical variables were reported as frequencies and percentages.

A comparative analysis was conducted to evaluate the demographic characteristics of both patients with non-traumatic spinal cord injury (SCI) (Group 1, *n* = 6) and patients with traumatic SCI (Group 2, *n* = 9). The primary variables assessed were age and gender. For the age comparison, the Mann–Whitney U test was employed due to the small sample sizes and the potential non-normal distribution of age data. This non-parametric test is appropriate for comparing the median ages between two independent groups when the data may not follow a normal distribution. For the gender comparison, the chi-square test was utilised to assess the association between group membership and gender distribution. This test is suitable for categorical data and determines whether there is a significant difference between the expected and observed frequencies in one or more categories. A *p*-value less than 0.05 was considered statistically significant for all tests. Statistical analyses were performed using IBM SPSS Statistics version 29.

## 3. Results

### 3.1. Demographic and Clinical Data

Data from 15 patients were included in the study. Patients’ average age was 59 years (SD 12.94, range 40–70), and the sample included two females. The median age of patients in the non-traumatic SCI group (Group 1) was 53 years (range: 49–59 years). The individual ages were 49, 52, 53, 53, 55, and 59 years. In the traumatic SCI group (Group 2), the median age was 55 years (range: 40–70 years), with individual ages of 40, 46, 54, 55, 55, 63, 65, 66, and 70 years. The Mann–Whitney U test indicated no statistically significant difference in age distribution between the two groups (U = 37, *p* = 0.43). This result suggests that the median ages of patients with non-traumatic and traumatic SCI are comparable.

Regarding gender distribution, the non-traumatic SCI group consisted of two females (33.3%) and four males (66.7%), while the traumatic SCI group consisted of one female (11.1%) and eight males (88.9%). The chi-square test revealed no significant association between group membership and gender distribution (χ^2^ = 1.111, df = 1, *p* = 0.29). This indicates that the proportions of males and females were similar in both groups. The comparative analysis demonstrated no significant differences in age or gender between patients with non-traumatic and traumatic SCI. Both groups are comparable in terms of these demographic variables, with *p*-values exceeding the conventional threshold of 0.05.

The severity of the spinal cord injuries varied, as classified by the AIS. In the traumatic SCI (tSCI) group, injuries ranged from ASIA A complete spinal cord injuries to ASIA D incomplete injuries. The causes of the spinal cord injuries were as follows: nine patients (60%) experienced traumatic injuries, including three patients classified as ASIA A tSCI. Non-traumatic causes encompassed one case of degenerative cervical myelopathy, two of neuropathic pain following thoraco-lumbar ependymoma resection, two cases of transverse myelitis-induced central neuropathic pain, and one ischemic spinal stroke case. A summary of patient demographics and implantation information can be found in Table 2 and Table 3.

Sleep disturbance was analysed by categorising patients into “Sleep Affected” or “Sleep Not Affected” based on their reported sleep issues. In the non-traumatic SCI group, 3 out of 6 patients (50%) reported sleep disturbances, while in the traumatic SCI group, 1 out of 9 patients (11.1%) reported sleep disturbances. A chi-square test was used to compare the prevalence of sleep disturbances between non-traumatic and traumatic SCI. The chi-square test statistic was 1.57, with a *p*-value of 0.210, indicating no significant difference in the frequency of sleep disturbances between the two groups.

Before an implant was fitted, trials were carried out on 9 individuals, while 5 patients went through a direct-to-implant procedure. The trial stimulation phase involved the temporary placement of electrodes near the spinal cord, connected to an external pulse generator. The goal was to assess whether the patient experiences sufficient pain relief, typically defined as a 50% or greater reduction in pain, before committing to a permanent implant. The trials lasted for two weeks, during which the patient would evaluate how well the SCS system managed their pain in daily life. If the trial was deemed successful, the patient proceeded to a permanent implantation of the SCS system.

In contrast, direct-to-implant procedures bypassed the trial phase, proceeding straight to permanent implantation of the leads and the pulse generator. This approach was selected when there was high confidence in the effectiveness of SCS for a particular patient, often based on prior response to similar interventions or when certain clinical factors strongly indicated that a trial may not be necessary.

**Figure 1:**
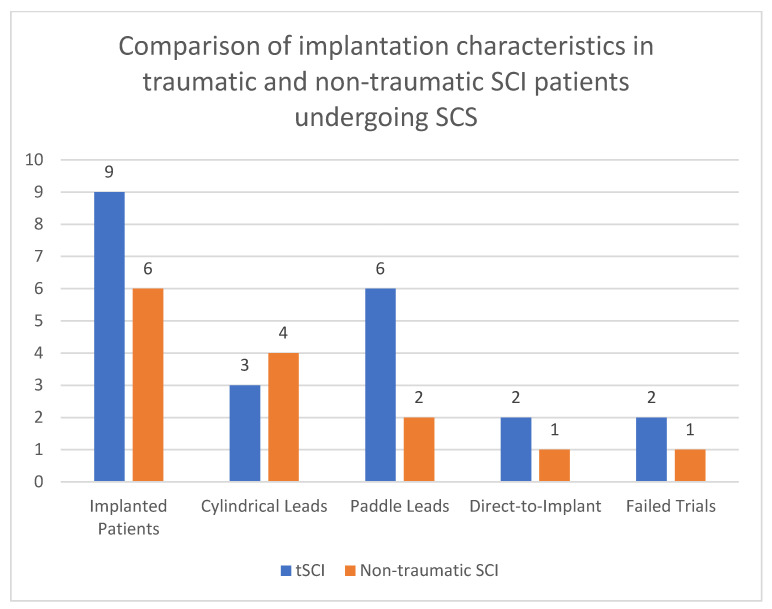
A chart delineating the subgroups of implanted patients (tSCI and non-traumatic SCI).

**Figure 2:**
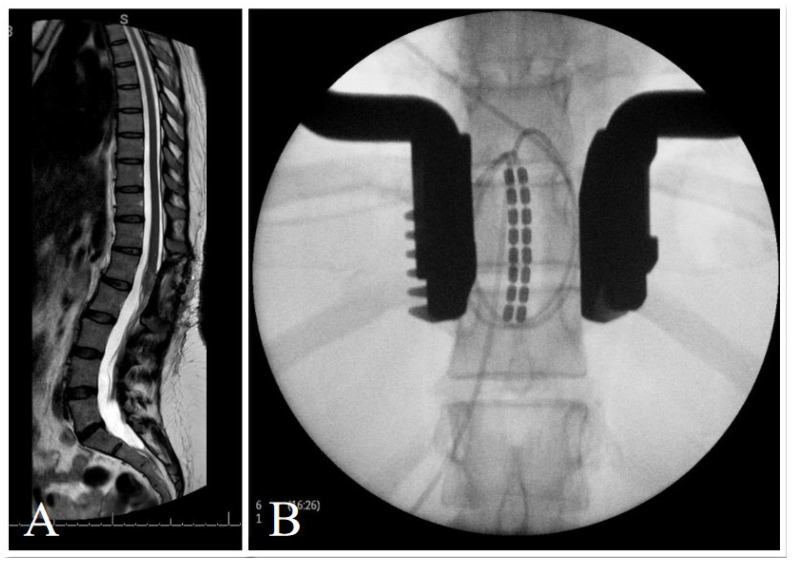
Retrograde paddle insertion at T9-10 for a case of post-ependymoma resection neuropathic pain in a 49-year-old female. Pre-operative MRI (**A**) and intra-operative X-ray (**B**). Boston Microburst was employed with a best anatomical location of T9.

**Figure 3:**
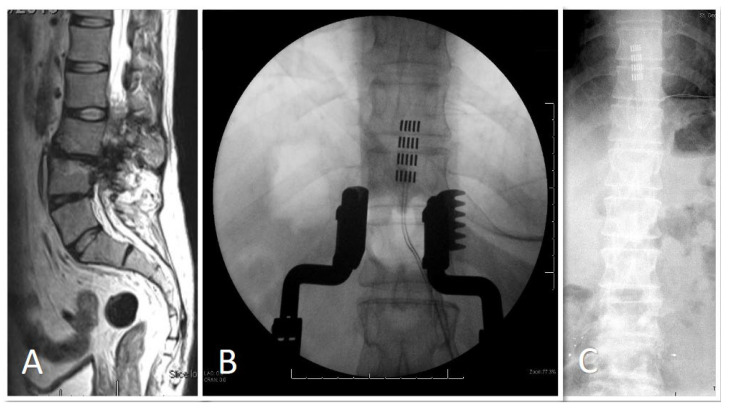
Paddle insertion at T10-11 for an ASIA B high-velocity trauma in a 40-year-old male. Pre-operative MRI (**A**), intra-operative X-ray (**B**), post-operative X-ray (**C**).

Across both groups, 3 patients (2 from the tSCI group and 1 from the non-traumatic group) underwent direct-to-implant procedures without trial stimulation (Figure 1). These patients had previously established neuropathic pain patterns that were deemed responsive to SCS. The remaining 12 patients underwent trial stimulation before permanent implantation, a standard procedure to assess the effectiveness of SCS in managing their specific pain patterns. Trial patients underwent an SCS trial period of 14 days. implanted and connected to an external stimulator device through an extension lead. If patients experienced a pain reduction ≥ 50% on the VAS a permanent implantable pulse generator (IPG) was placed during surgery. IPGs were sited in the lateral thoracic wall and connected to the stimulator leads. Post-operatively, device settings (i.e., bipolar configuration, stimulation frequency, pulse width) were individually programmed in conjunction with device representatives in order to achieve optimal pain relief for the patient. If trial stimulation was unsuccessful, the lead was removed. Electrodes implanted were either cylindrical (7 patients) or paddle (8 patients) electrodes. Their placements within the spinal canal varied from levels C2 to T11 (Figure 2 and Figure 3). SCS implants were from different manufacturers, including Nevro^TM^, Boston Scientific^TM^ and Abbott^TM^.

Stimulation parameters in permanently implanted patients included a high-frequency protocol [23] (10 kHz) in 5 patients, a paraesthesia programme in 2 patients, Abbott Burst DR^TM^ in 3 patients [24,25], Boston Microburst^TM^ in 1 patient and Boston FAST^TM^ therapy [26] in 1 patient. These therapies offered alternative burst and frequency-modulation techniques aimed at optimising pain management while minimising energy consumption. High-frequency stimulation (10 kHz) delivers rapid electrical pulses without eliciting paraesthesia. The absence of paraesthesia allows for uninterrupted pain management, making it a favourable option for many patients. Abbott’s BurstDR™ mimics neural activity by delivering electrical bursts that modulate pain signals without causing constant sensory stimulation. MicroBurst™ therapy involves delivering shorter bursts of stimulation with fewer pulses, aimed at conserving battery life while still providing effective pain relief. Finally, FAST therapy is designed to address both neuropathic and nociceptive pain, making it a versatile option for patients with mixed pain syndromes.

### 3.2. Pain Relief Outcomes

Of the three ASIA A patients, two failed their trials and one was successful, with one being a paddle and one being percutaneous. For the 7 successfully treated tSCI patients, a decrease from 8.6 to 4.5 in mean VAS was noted following SCS implantation. Of these, 71% reported experiencing more than 50% relief from pain, classifying them as a successful SCS outcome. Moreover, an increased EQ-5D score from −0.133 to 0.165 was observed. The remaining patients also experienced reductions in pain, though less significant, suggesting that additional modulation of SCS parameters may be required.

In the 5 successfully treated non-traumatic SCI patients, a substantial decrease in mean VAS scores from 8.5 to 2.5 was observed, with all of them (100%) reporting more than 50% relief in pain. Their mean EQ-5D scores saw substantial improvement post-SCS implantation, with the scores rising from 0.209 to 0.713.

The pain severity, as assessed by the NRS, was compared between patients with non-traumatic and traumatic SCI using the Mann–Whitney U test. The median NRS score for non-traumatic SCI was 7 (ranging from 7 to 9), while the median VAS score for traumatic SCI was 8 (ranging from 7 to 9). The Mann–Whitney U test statistic was 4.5, and the corresponding *p*-value was 1.0, indicating no significant difference in pain severity between non-traumatic and traumatic SCI. This suggests that both groups experience similar levels of pain severity.

### 3.3. Pain Medication Change Outcome

In 49% of the cohort, an overall reduction in medication usage was described, including anti-neuropathic medications such as pregabalin and gabapentin and opioid analgesia. Only one patient was reported to have an increase in analgesia medication due to incomplete coverage of one of their pain regions. However, 49% of the cohort had complete data regarding their medication changes due to supra-regional referral to our centre.

### 3.4. Adverse Events

One adverse event was recorded leading to a 7% occurrence within the cohort prompting a revision of the generator site due to seroma formation. This patient required a revision surgery due to seroma formation at the generator site. The complication was managed successfully with the revision, and the patient continued to benefit from the SCS system post-revision. No other major complications, such as infections, lead migration, or hardware failure, were observed during the 12-month follow-up period.

## 4. Discussion

This study compared pain severity, functional mobility, sleep disturbances, and treatment outcomes between patients with non-traumatic and traumatic spinal SCI who underwent SCS. In terms of SCS outcomes, patients with non-traumatic SCI demonstrated greater improvements in pain relief and quality-of-life scores compared to those with traumatic SCI. Non-traumatic SCI patients reported 100% success in achieving more than 50% pain relief, while 71% of traumatic SCI patients experienced similar relief. A reduction in pain medication usage was observed in 49% of the cohort that had this data available, with minimal adverse events reported.

Neuropathic pain following SCI is among the most complex and challenging pain syndromes. The mechanisms underlying this pain involve significant anatomical and functional changes near the injury site in the spinal cord. While early studies focused on the spinal cord as the primary focal point, recent animal models have revealed additional sites contributing to neuropathic pain after SCI [27,28,29,30,31].

Acute ischaemic or traumatic damage initiates a series of interconnected and self-sustaining events within the spinal cord, including anatomical, neurochemical, excitotoxic, and inflammatory alterations [17,32,33,34,35,36,37,38,39,40,41]. These factors lead to changes in spinal neuron function, resulting in pain. This cascade does not occur in a fixed sequence and is influenced by factors such as sex, genetic strain, gonadal hormones, and selective neuroprotective agents. Inflammation involving cytokines, prostaglandins, and reactive oxygen species, along with neuromodulatory agents like glutamate, GABA, opioids, and monoamines, alter neuronal expression and function [35,40,41,42,43,44,45,46,47,48]. This results in the activation of microglia and astrocytes, altered neuronal firing—including increased neuron recruitment, irregular background activity, and changes in sodium currents—and long-term synaptic plasticity, which encompasses modified synaptic connections, regulatory proteins, neuronal survival or apoptosis, and altered gene expression [35,40,41,42,43,44,45,46,47,48].

SCS has emerged as a promising alternative solution for addressing the pain associated with SCI [23]. It involves the implantation of a small device near the spinal cord that delivers mild electrical pulses to the dorsal columns, intercepting and modulating the pain signals before they reach the brain [49]. SCS provides analgesic effects in patients with SCI by modulating pain pathways, a mechanism that can be understood through the gate control theory proposed by Melzack and Wall in 1965 [50]. This theory suggests that pain signals transmitted by small nerve fibres (A-delta and C fibres) can be inhibited at the spinal cord level by activating larger, non-pain-transmitting fibres (A-beta fibres). When these larger fibres are stimulated, they “close the gate” in the dorsal horn of the spinal cord, reducing the transmission of pain signals to the brain.

In the context of SCI patients, SCS may work by activating inhibitory pathways. Electrical impulses are delivered to the dorsal columns of the spinal cord, which are rich in large-diameter afferent fibres. By stimulating these fibres, SCS enhances inhibitory signals that suppress the activity of pain-transmitting neurons. Neurophysiological studies, such as those by Larson et al., have demonstrated that dorsal column stimulation alters neuronal firing patterns in both humans and monkeys, correlating with pain relief [51]. This suggests that SCS may modify the processing of pain signals at the spinal level. Furthermore, SCS inhibits nociceptive neurons. Research by Dubuisson found that dorsal column stimulation affects neurons in the substantia gelatinosa and marginal zone of the spinal cord—key areas involved in pain transmission [52]. By inhibiting the activity of these nociceptive neurons, SCS may reduce pain sensation. In SCI patients, there is often hyperexcitability of spinal neurons below the injury level. SCS may suppress this abnormal activity, alleviating neuropathic pain symptoms.

SCS also induces supraspinal interactions. Bantli et al. demonstrated that dorsal column stimulation can affect pain modulation pathways in the brain [53]. This means that SCS not only acts at the spinal level but also influences central pain processing mechanisms. The gate control theory remains relevant as it provides a framework for understanding how stimulation of non-painful fibres can inhibit pain signals. SCS may utilise this principle by activating large-diameter fibres to “close the gate” on pain transmission. Additionally, SCS may promote the release of inhibitory neurotransmitters, such as GABA and endogenous opioids, further enhancing its analgesic effects [54,55,56,57].

This retrospective study demonstrates that SCS is effective in alleviating neuropathic pain, enhancing quality of life, and reducing dependence on opioid medications and other analgesics in patients with spinal cord injury. These data demonstrate the potential of spinal cord stimulation, particularly high-frequency and burst-based protocols, in providing significant pain relief and quality-of-life improvements for both traumatic and non-traumatic spinal cord injury patients. The prevalence of chronic neuropathic pain in patients with SCI is a significant concern, affecting up to 85% of individuals with SCI [58]. Traditional pain management techniques, such as medications, physical therapy, and nerve block injections, often provide limited relief, if at all [59]. A recent retrospective, single-centre observational study investigated its impact on healthcare utilisation (HCU) [60]. The study involved 160 subjects who received a high-frequency (10 kHz) SCS implant. HCU trends were assessed by measuring opioid consumption in morphine milligram equivalents and monitoring emergency department (ED) visits and outpatient visits for interventional pain procedures during the 12 months before and after the SCS implantation. Impressively, 91.5% of patients achieved a minimally clinically important difference in opioid reduction. There was also a significant decrease in ED visits, from a mean of 0.12 pre-implant to 0.03 post-implant (*p* < 0.01), and a reduction in office visits for interventional pain procedures from 1.39 to 0.28 (*p* < 0.0001).

Several factors contribute to the growing interest in using SCS for SCI pain management [23,49]. Firstly, the modulatory effect of SCS on the spinal circuits is unknown but thought to counteract the central sensitisation induced by SCI, providing pain relief [49,61]. Secondly, SCS is a minimally invasive technique that is reversible, and its efficacy can be assessed during a trial period before committing to the implant. Moreover, SCS devices can be programmed and adjusted to optimally manage pain in each individual patient.

SCS treatment in ASIA A SCI patients has previously not been indicated. Given that ASIA A patients are characterised by a complete absence of both motor and sensory functions below the level of the lesion, one would not expect SCS to be effective for such patients [62]. However, within our cohort of three ASIA A patients, one patient was implanted successfully and achieved a 70% reduction in their pain with SCS treatment. One of the principal hypotheses behind the efficacy of SCS is its potential to modulate latent but still viable neural circuits that remain post-injury [63]. By delivering electrical impulses through surgically implanted electrodes to specific regions of the spinal cord, SCS can facilitate the transmission of neuronal signals across damaged segments, thereby enhancing residual motor and sensory pathways [64]. Studies have indicated that when SCS is paired with intensive rehabilitative therapies, the synergistic effect can promote neuroplasticity, thus optimising the potential for functional recovery [65]. Although the exact mechanisms underlying these outcomes require further elucidation, the preliminary evidence suggests that SCS presents an innovative approach for not only functional restoration in patients with ASIA A spinal cord injuries but also for the treatment of SCI-related neuropathic pain.

The comparison of our study with the findings of Sokal et al. [66] underscores both the potential and the limitations of spinal cord stimulation in managing central neuropathic pain (CNP) following spinal cord injury. Their case series involved eight SCI patients who were treated with either burst or high-frequency stimulation paradigms. Over a one-year follow-up period, only two patients experienced significant pain relief, indicating that these newer stimulation techniques had limited success in alleviating CNP in the majority of patients.

In our patient group, traumatic SCI patients showed a mean reduction in VAS scores from 8.6 to 4.5, and non-traumatic patients from 8.5 to 2.5, with substantial pain relief observed in 66% of traumatic and 100% of non-traumatic cases. This contrasts with the findings of Sokal et al., where the majority of patients did not show significant improvement, highlighting the ongoing challenge of treating CNP with SCS. Despite employing advanced stimulation paradigms such as burst and high-frequency SCS, Sokal et al. found no significant superiority over traditional methods. It is worth noting that the authors employed 1.2 kHz high-frequency stimulation. This is notably lower than the more common high-frequency setting of 10 kHz employed in some of our patient group, which is typically used to deliver paraesthesia-free pain relief.

However, both studies highlight the variability in patient outcomes and the critical need for careful patient selection. SCS appears to be more effective in patients with incomplete SCI and preserved motor and sensory functions, as noted by Sokal et al. The adverse event rate was low in both studies, with our study reporting a single case of seroma formation requiring revision surgery.

Previous studies, such as those by Kapural et al. and Taylor et al., have demonstrated that SCS can achieve significant pain relief, with approximately 60–76% of patients experiencing at least a 50% reduction in pain scores [67,68]. Quality-of-life improvements, as measured by tools like the EQ-5D, have also been reported, showcasing the functional benefits of SCS in chronic pain management. In comparison, the findings from our study show an 86.7% success rate for permanent SCS implantation, with notable reductions in pain scores and significant improvements in quality-of-life metrics. This success rate exceeds many of the benchmarks in the existing literature and suggests that the use of high-frequency SCS, such as 10 kHz, may contribute to these enhanced outcomes [69,70,71,72]. The alignment of our results with the upper range of previously reported success rates underscores the potential advantages of modern, high-frequency SCS protocols in effectively managing SCI-related pain.

The topic of conducting trials versus direct implant for SCS patients is a subject of ongoing debate [73,74]. Considering the extensive anatomical and pathological alterations that occur following SCI, it becomes essential to adapt SCS treatment individually for each patient. This personalised approach is guided by the patient’s remaining neuronal structures and current functional status, aiming to achieve the most favourable therapeutic outcome. In our group, 11 patients (79%) were given trials since the efficacy of direct implantation SCS devices for their pathology was uncertain. In a study examining the optimal duration of trial periods for SCS, 40 patients underwent an SCS trial and were questioned daily about their willingness to proceed to a permanent implant [75]. A successful trial was determined by three consecutive affirmative responses, while three negative replies indicated a failed trial. Patients also provided daily ratings of surgical pain, original pain, satisfaction with the stimulator, and the duration of stimulator use. The results revealed that trial durations ranged from 3 to 15 days. Patients who ultimately had unsuccessful trials took longer to decide and experienced prolonged surgical pain. In contrast, those with successful trials often reported more than a 50% reduction in pain and made quicker decisions to proceed. Notably, the initial infection rate was 7.5%, which decreased to 2.8% after the implementation of an improved dressing protocol, underscoring the importance of safety measures. The study concluded that all patients could make an informed decision within 15 days, with successful trials requiring less time.

Complications associated with SCS are numerous, with some studies reporting incidence rates as high as 30% to 40% [76]. Hardware-related issues, such as lead failure and migration, are more frequently encountered compared to infection, pain, and wound breakdown. Infection is a significant concern, occurring in approximately 3.4% to 10% of cases, and is one of the leading reasons for device removal and therapy failure. Importantly, the rate of complications in SCS is notably influenced by the experience and expertise of the clinician performing the implantation [76].

The selection of stimulation modalities was not uniform for all patients. They were provided with devices that had the most alignment with their pain distribution as well as preference for primary cell or rechargeable devices. As there is a wide variety of manufacturers, programming the SCS devices varied based on the device maker’s protocols. Despite this, many patients were treated with HF SCS. This is because the effectiveness of conventional SCS for treating SCI pain and its long-term results are still sub-optimal. Typically, individuals with SCI do not experience the same level of relief from this method as do those suffering from complications of failed back surgeries or neuropathic pain with a peripheral cause [61].

### Limitations

An important outcome that aids in understanding the overall effectiveness of SCS for SCI pain is the reduction in pain medication. In this study, 49% of patients reported a reduction in medication usage; however, data were incomplete for 51% of the patient group due to the supra-regional nature of patient follow-up. To maintain transparency, we acknowledge that conclusions regarding medication reduction are drawn from the subset of patients with complete data. This limitation underscores the need for cautious interpretation of these results and highlights the importance of future studies employing methods to address missing data, such as imputation techniques, for a more comprehensive analysis.

We also recognise that our study is limited by the small sample size, which restricted our ability to conduct further subgroup analyses or draw definitive conclusions from these comparisons.

While our outcomes are encouraging, the retrospective nature of the study and potential biases related to patient selection and record completeness warrant further investigation in future prospective, randomised trials to validate these findings and establish more robust clinical guidelines. The study also presents other limitations, including the heterogeneity of the patient population, different causes and levels of spinal cord injuries, the variation in the type of devices used, and the sample size itself.

Challenges and areas requiring further research include identifying the optimal parameters of SCS for different pain types and SCI aetiologies, understanding the long-term effects and safety of SCS, and enhancing the predictability of SCS outcomes through adequately powered and controlled studies.

## 5. Conclusions

HF SCS can be effective option for managing traumatic and non-traumatic SCI-related pain, offering advancements over traditional methods. The significant reductions in pain intensity observed in patients, as well as improvements in functional outcomes and quality of life, highlight the advantages of HF SCS over traditional pain management methods. The observed reduction in reliance on pharmacological interventions, including opioid and neuropathic pain medications, further underscores the potential of HF SCS to improve patient outcomes and reduce the burden of chronic pain management. Despite these positive outcomes, challenges remain in optimising SCS therapy for SCI patients. Variability in individual patient responses, the technical complexity of device implantation, and the need for precise programming to achieve optimal pain relief are areas that require ongoing attention. Moreover, long-term data on the durability of pain relief and device functionality are necessary to fully establish the benefits and limitations of HF SCS.

## Figures and Tables

**Table 1:** Summary of the ASIA scale, categorising patients based on the degree of motor and sensory function preserved below the level of the spinal cord injury.

ASIA Classification	Description	Motor Function	Sensory Function
A (Complete)	No motor or sensory function is preserved below the level of injury, including in the sacral segments (S4–S5).	No motor function below the injury level.	No sensory function below the injury level.
B (Sensory Incomplete)	Sensory but not motor function is preserved below the level of injury, including the sacral segments (S4–S5).	No motor function below the injury level.	Sensory function preserved below injury level.
C (Motor Incomplete)	Motor function is preserved below the level of injury, but more than half of key muscles have strength <3.	Weak motor function (less than grade 3).	Sensory function may or may not be preserved.
D (Motor Incomplete)	Motor function is preserved below the level of injury, and at least half of key muscles have strength ≥3.	Strong motor function (grade 3 or higher).	Sensory function may or may not be preserved.
E (Normal)	Normal motor and sensory function are preserved below the level of injury.	Normal motor function below injury level.	Normal sensory function below injury level.

**Table 2:** Non-TSCI patient demographics and implantation details.

Age	Sex	Indication	Percutaneous/Paddle	Manufacturer	Program	Anatomical Location of Best Program
49	F	Ependymoma	Retrograde paddle T9–10	Boston (Marlborough, MA, USA)	Microburst	T9
55	M	Transverse myelitis	Percutaneous	Boston	FAST	T3
59	M	Transverse Myelitis C2–C5 + FBSS L4/L5	Percutaneous T8–9	Nevro (Redwood City, CA, USA)	HFX	T8
53	M	Cervical myelopathy	Percutaneous C2–6	Abbott (Green Oaks, IL, USA)	BurstDR	C2
52	M	Spinal stroke T3	Paddle T9	Abbott	BurstDR	T9–10

**Table 3:** TSCI patient demographics and implantation details.

Age	Sex	Indication	Percutaneous/Paddle	Manufacturer	ASIA	Program	Anatomical Location of Best Program
55	M	T8 SCI	Percutaneous	Nevro	D	HFX	
66	M	T10 to L5 spinal fusion procedure	Paddle T7 to T10	Nevro	C	HFX	Mid T9
65	M	T8 SCI	Paddle over T2	Abbott	A	Paraesthesia	Mid T2
40	M	L1 SCI	Paddle T11	Abbott	C	BurstDR	T10/T11
70	M	L3 SCI	Paddle T10–T11	Abbott	D	BurstDR	T10/11
54	M	C4 SCI	Paddle C4–C7	Nevro	B	HFX	High C3
46	F	T3 SCI	Percutaenous	Nevro	A	HFX	

## Data Availability

The datasets presented in this article are not readily available because the data are part of an ongoing study. Requests to access the datasets should be directed to A.A.
